# Experimental 3-D Ultrasound Imaging with 2-D Sparse Arrays using Focused and Diverging Waves

**DOI:** 10.1038/s41598-018-27490-2

**Published:** 2018-06-14

**Authors:** Emmanuel Roux, François Varray, Lorena Petrusca, Christian Cachard, Piero Tortoli, Hervé Liebgott

**Affiliations:** 1 Department of Information Engineering, University of Florence, Firenze, Italy; 2 Univ Lyon, INSA-Lyon, Université Claude Bernard Lyon 1, UJM-Saint Etienne, CNRS, Inserm, CREATIS UMR 5220, U1206 F-69621 Lyon, France; 3 Univ Lyon, UJM-Saint-Etienne, INSA, CNRS UMR 5520, INSERM U1206, CREATIS, F-42023 Saint-Etienne, France

## Abstract

Three dimensional ultrasound (3-D US) imaging methods based on 2-D array probes are increasingly investigated. However, the experimental test of new 3-D US approaches is contrasted by the need of controlling very large numbers of probe elements. Although this problem may be overcome by the use of 2-D sparse arrays, just a few experimental results have so far corroborated the validity of this approach. In this paper, we experimentally compare the performance of a fully wired 1024-element (32 × 32) array, assumed as reference, to that of a 256-element random and of an “optimized” 2-D sparse array, in both focused and compounded diverging wave (DW) transmission modes. The experimental results in 3-D focused mode show that the resolution and contrast produced by the optimized sparse array are close to those of the full array while using 25% of elements. Furthermore, the experimental results in 3-D DW mode and 3-D focused mode are also compared for the first time and they show that both the contrast and the resolution performance are higher when using the 3-D DW at volume rates up to 90/second which represent a 36x speed up factor compared to the focused mode.

## Introduction

Both academic and industrial research groups are thoroughly investigating new methods to develop real-time 3-D ultrasound (3-D US) imaging^1–3^. Real-time 3-D US imaging would reduce the (operator dependent) diagnosis variability and improves the estimation accuracy by accessing directly to volumetric dimensions (organs or tumors) and avoiding physiological measurements to be elaborated from distinct planes measurements. In particular, there are very high expectations for real-time 3-D echocardiography which is one of the most challenging US applications^4–7^ because it requires very high spatio-temporal resolution, i.e. high volume rates, while maintaining fair accuracy and robustness in the spatial domain.

Before the development of electronic steering applied to linear (1-D) arrays^8^, first 2-D images were obtained by producing each line by means of a single-element transducer that was manually or mechanically translated. The same trend was observed in first 3-D imaging experiments, which actually used position-tracked (free-hand) scanning^9–12^ or motorized 1D arrays that were translated^13,14^, rotated^15–17^, or tilted^18,19^ to scan a volume (mechanical scanning). The introduction of 1.25 D, 1.5 D and 1.75 D arrays, described in^20,21^ has paved the way to 2-D arrays, which extend the possibility of obtaining full electronic apodization, focusing and steering over an entire volume.

Different strategies to design and drive 2-D arrays have been investigated. Ideally, in a full 2-D array, each element should be continuously driven by the scanner. This solution requires as many scanner channels as the number of elements (e.g. 1024 for a 32 × 32 array), which for now is only achieved using cumbersome hardware available in few research centers (the SARUS scanner at the Technical University of Denmark in Lyngby^22^, the parallelized Aixplorer systems at the Langevin Institute in Paris^23^, which was also used in 3-D US ultrafast imaging experiments^4^, the parallelized Verasonics systems at the University of Lyon^24^). On the other hand, promising techniques allow addressing a large amount of active elements with a reduced number of channels: micro-beamforming^25–33^, row-column addressing (RCA)^34–39^, and channel multiplexing^40^. However, the acquisition flexibility is reduced because of the pre-allocated delays associated to the sub-arrays (in the micro-beamforming strategy) or because the elements are not continuously connected to the scanner (when using RCA or multiplexing). The cost and the power dissipation of the embedded electronics, as well as the skills and the time required to program specific sequences on such integrated circuits, make them less convenient for research tests, and unsuitable for low-cost equipment.

By contrast, sparse arrays can be designed to have a number of active elements equal to the available number of channels, which allows a continuous one-element-to-one-channel connectivity, preserving full flexibility in elements driving. For a desired number of active elements, 2-D sparse arrays can be optimized^41–46^ to produce homogeneous imaging capability over the entire volume of interest. In^41,47^, in particular, a simulated-annealing-based 2-D sparse array optimization framework was developed and the performance evaluated by wideband acoustic simulations. However, compared to full arrays, the sensitivity of 2-D sparse arrays is lowered by the reduced active surface, and this has probably so far discouraged the intensive development of this type of probes, which were experimentally validated only in a few studies limited to focused transmission mode^32,33,48,49^.

In order to facilitate the experimental test of different sparse array configurations, the optimization tool described in^41,47^ was used to find the best combination of 256 active elements (opti256) out of a commercial 32 × 32 fully populated array (ref1024)^47^. First experimental imaging results obtained in focused and diverging wave (DW) transmission modes were presented in^50^ and^51^, respectively. In this study, extensive experimental 3-D US imaging results obtained with opti256 and a 256-element random sparse array (rand256) are shown. The related contrast/resolution performance is found to be sufficiently competitive when compared with that of the reference array. Finally, it is shown for the first time that a compounding strategy^52–59^ applied to DW transmission allows to significantly enhance the contrast and resolution of the 3-D US images with respect to 3-D focused mode, while offering a higher volume rate.

The paper is organized as follows. Section II.A shows the results of comparison among different 3-D focused mode acquisitions, and section II.B shows the results of comparison among different 3-D DWs acquisitions. The results are discussed in section III. Section IV.A introduces the used 2-D arrays configurations, while section IV.B details their practical implementation. The experimental set-up implemented to acquire 3-D focused US and 3-D DW US images, is illustrated in section IV.C.1 and section IV.C.2, respectively.

## Results

### Focused mode

**Figure 1:**
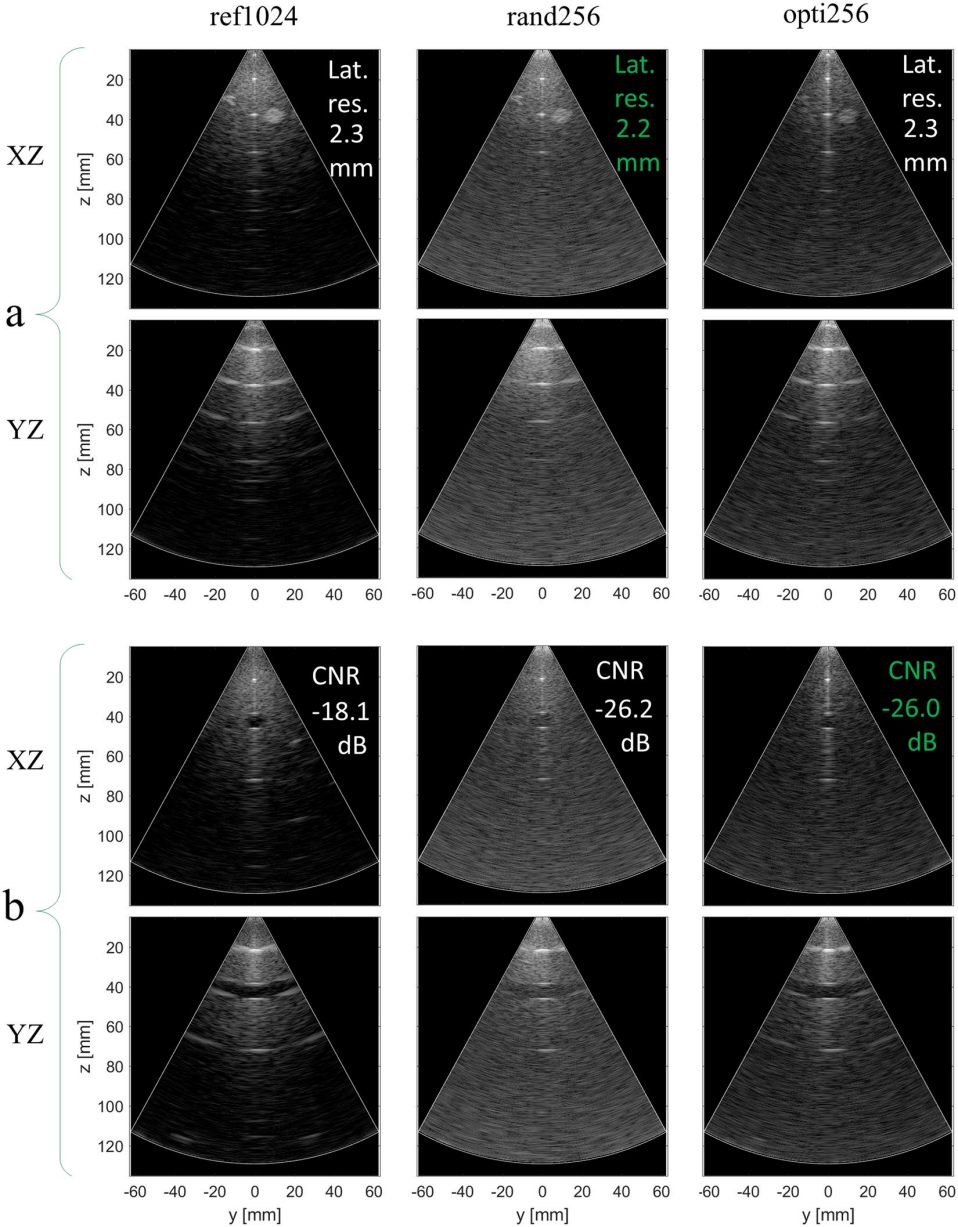
Phantom 3-D focused image slices for (**a**) resolution and (**b**) contrast evaluation comparison between the random array (rand256 on the right-hand column), the optimal sparse array opti256 central columns), and the reference array (ref1024 on the left-hand column). The best performance between the two sparse arrays is highlighted in green. The dynamic range is 60 dB. XZ and YZ refer to the central image planes in the azimuth and elevation direction, respectively.

**Figure 2:**
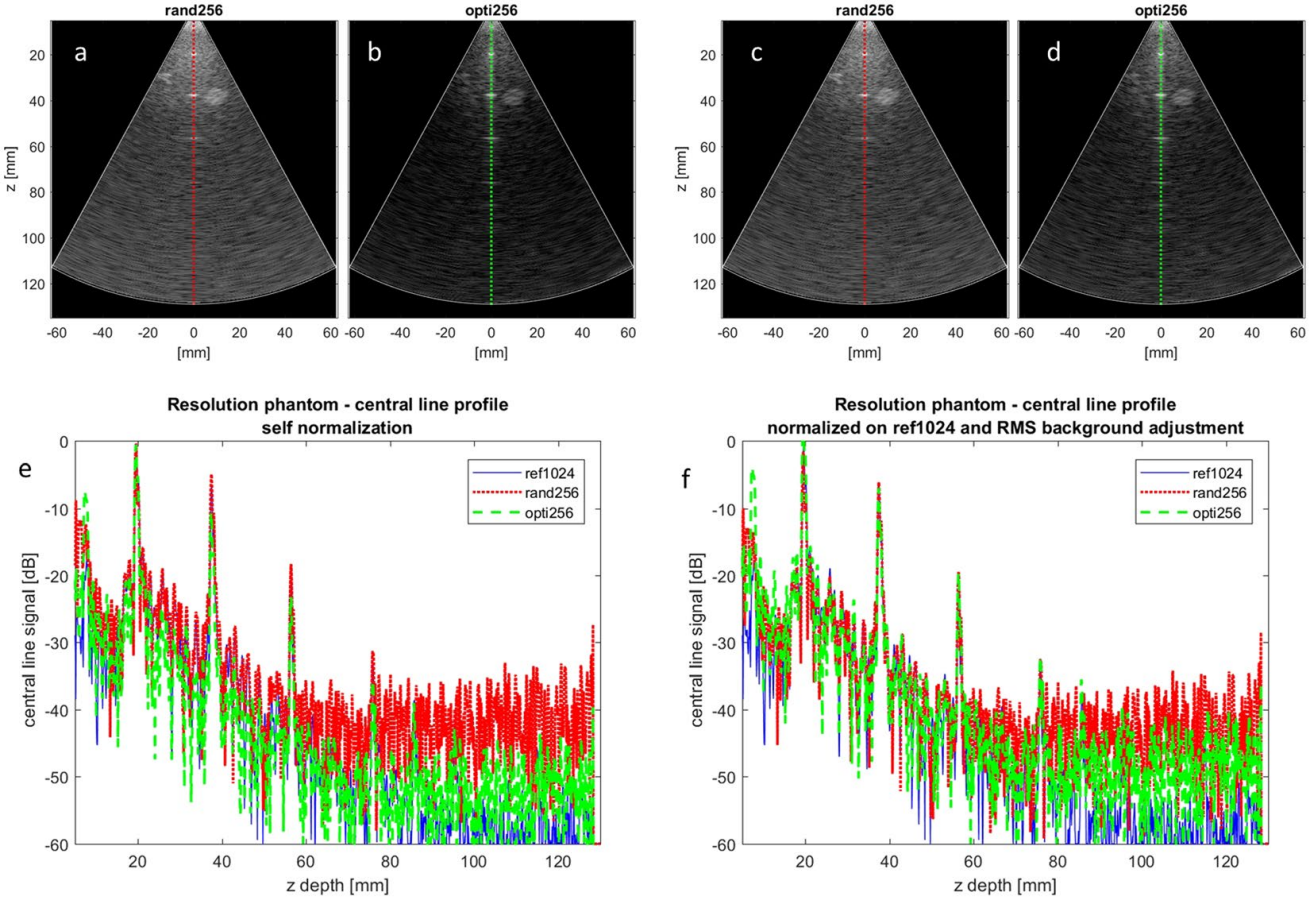
(**a**,**b**) XZ slices (normalized on their own maximum) of the 3-D data backscattered by the CIRS (054GS) phantom when the rand256 and opti256 arrays were used in focused mode. (**c**,**d**) The background RMS adjustment of (**a**) and (**b**). (**e**) The central line profiles of the XZ slice resolution phantom images without the RMS background adjustment: the RMS background values are very different in particular for rand256. (**f**) The adjustment to make the RMS background value of rand256 and opti256 images match with the one obtained on the image of ref1024. The image dynamic range is 60 dB.

The XZ and YZ slices of the 3-D images obtained in focused mode with the three arrays (ref1024, rand256 and opti256) are displayed in Fig. 1. As detailed in Fig. 2, the 3-D focused images produced with rand256 performed very badly in terms of contrast when normalized on their own maximum value (the origin of this sensitivity reduction is discussed in III). To make the images produced by all the three arrays qualitatively comparable they were all normalized on the maximum of ref1024 and adjusted so that the average background RMS value (evaluated on the central line of the resolution phantom images) is the same for all arrays.

To compare opti256, rand256 and ref1024, quantitative resolution and contrast measurement results are presented in Table 1. In terms of lateral resolution rand256 yields the best performance with 2.2 mm (on average), which is just 0.1 mm better than with the reference and opti256. The opti256 array yields the same average lateral resolution (2.3 mm) as the reference array, which validates that the optimization process efficiently shaped the main lobe of the opti256 beam profile. In terms of axial resolution, both ref1024 and rand256 arrays yield 0.5 mm on average, while opti256 yields around 0.6 mm on average.

**Table 1:** Evaluated resolution and contrast for the optimized, the random and the reference arrays in focused mode.

Resolution (mm)	ref1024	rand256	opti256
lateral 20 mm	1.4	1.7	1.2
lateral 40 mm	2.3	2.0	2.3
lateral 60 mm	3.3	2.8	3.4
**Average lat. resolution**	2.3	2.2	2.3
axial 20 mm	0.6	0.5	0.9
axial 40 mm	0.5	0.5	0.6
axial 60 mm	0.5	0.5	0.5
**Average ax. resolution**	0.5	0.5	0.7
**Contrast**	**ref1024**	**rand256**	**opti256**
CR (dB) ROI 1	−8.5	−1.4	−2.1
CR (dB) ROI 2	−8.2	−2.5	−0.9
CR (dB) ROI 3	−9.8	−3.0	−6.7
**Average CR**	−8.8	−2.3	−2.9
CNR (dB) ROI 1	−18.6	−30.7	−30.2
CNR (dB) ROI 2	−19.0	−25.2	−38.0
CNR (dB) ROI 3	−16.7	−24.1	−19.4
**Average CNR**	−18.1	−26.2	−26.0
sSNR ROI 1	2.2	2.2	2.5
sSNR ROI 2	2.1	2.3	2.6
sSNR ROI 3	2.4	1.9	2.2
**Average sSNR**	2.2	2.1	2.4

The best/worst performance are highlighted in green/red on each average line.

In terms of contrast, the images produced by the opti256 and ref1024 arrays look similar (Fig. 1). Quantitatively, the CR and the CNR reported in Table 1 follow the same trend: the best performance is given by ref1024 (CR = −8.8 dB and CNR = −18.1 dB), while the opti256 array yields 5.9 dB higher CR values and 7.9 dB lower CNR values, respectively. Finally, the rand256 array contrast performance is worse than opti256 by 0.6 dB and 0.2 dB in terms CR and CNR, respectively. The speckle variation indicated by the sSNR metric is similar for the three arrays, which consequently present equivalent background texture (it is a little better with opti256).

To summarize, the results support the conclusion that opti256 presents the same resolution performance as the full array and only about 6.0 dB and 8.0 dB of CR and CNR loss, while using 25% of the active elements. Moreover, the comparison between opti256 and rand256 experimentally confirms that the optimized 2-D sparse configuration yields an improvement trade-off between resolution and contrast for the focused 3-D US images.

### Diverging wave mode

**Figure 3:**
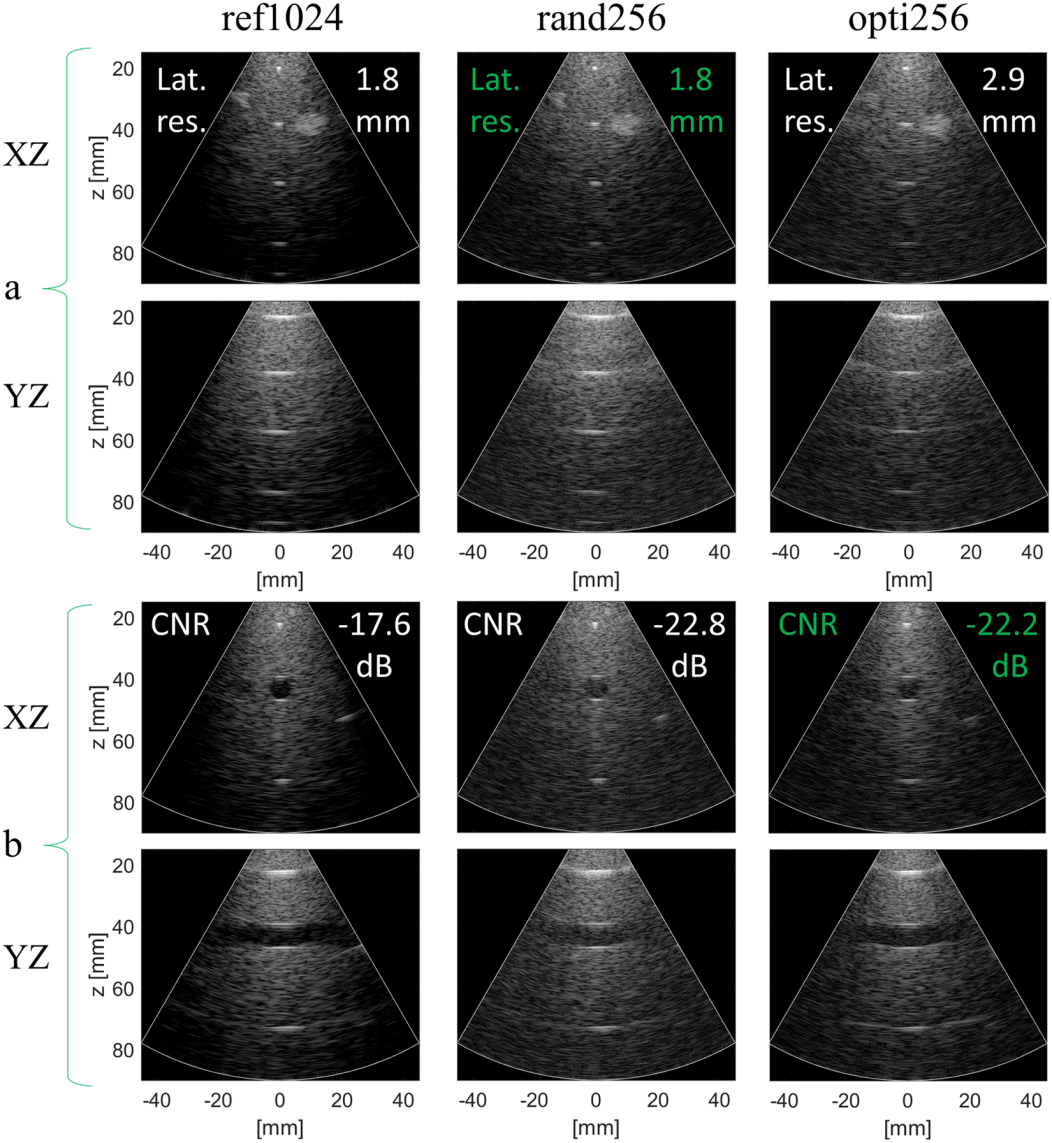
Phantom 3-D diverging wave image slices for (**a**) resolution and (**b**) contrast evaluation comparison between the reference array (ref1024 on the left-hand column), the random array (rand256 central column), and the optimal sparse array opti256 on the right-hand column). The best performance between the two sparse arrays is highlighted in green. The dynamic range is 60 dB. XZ and YZ refer to the central image planes in the azimuth and elevation direction respectively.

The XZ and YZ slices of the 3-D data volumes shown in Fig. 3 demonstrate the feasibility of performing 3-D ultrafast US imaging by transmitting DWs through 2-D sparse arrays. The quality of such images was assessed, and the quantitative resolution and contrast results are presented in Table 2.

**Table 2:** Evaluated resolution and contrast for the optimized, the random and the reference arrays in diverging mode.

Resolution (mm)	ref1024	rand256	opti256
lateral 20 mm	1.0	1.0	1.4
lateral 40 mm	1.9	1.9	3.1
lateral 60 mm	2.4	2.5	4.3
**Average lat. resolution**	1.8	1.8	2.9
axial 20 mm	0.8	0.7	0.4
axial 40 mm	0.5	0.5	0.4
axial 60 mm	0.8	0.5	0.4
**Average ax. resolution**	0.7	0.6	0.4
**Contrast**	**ref1024**	**rand256**	**opti256**
CR (dB) ROI 1	−9.6	−3.1	−4.2
CR (dB) ROI 2	−9.8	−4.6	−5.2
CR (dB) ROI 3	−11.0	−4.4	−5.1
**Average CR**	−10.1	−4.0	−4.8
CNR (dB) ROI 1	−18.8	−25.8	−24.0
CNR (dB) ROI 2	−18.1	−21.6	−21.6
CNR (dB) ROI 3	−16.0	−21.7	−21.4
**Average CNR**	−17.6	−22.8	−22.2
sSNR ROI 1	1.6	1.7	1.7
sSNR ROI 2	1.9	2.0	1.9
sSNR ROI 3	2.4	2.4	2.4
**Average sSNR**	2.0	2.0	2.0

The best/worst performance are highlighted in green/red on each average line.

In terms of lateral resolution, the best results are given by the ref1024 and rand256, with a 1.8 mm average resolution for the three wires. The opti256 array shows a coarser lateral resolution performance (2.9 mm). In terms of axial resolution, the arrays are ranked in a reversed order: the best performance (0.4 mm) is given by opti256 followed by rand256 (0.6 mm). The worst axial resolution performance is yielded by ref1024 (0.7 mm).

In terms of contrast, the XZ and YZ images produced by both opti256 and rand256 in 3-D DW mode are similarly good, as the anechoic cyst at 40 mm depth is clearly visible as in the image produced by the ref1024. Yet, the inside of the cyst is a little bit darker for opti256 compared to rand256, in particular on the YZ plane where the borders are more visible with opti256. Quantitatively (Table 2), the best results for contrast are given by the ref1024 array with a CR of −10.1 dB and CNR of −17.6 dB. The CR and CNR of opti256 are respectively 5.3 dB and 4.7 dB worse than with ref1024. Compared to rand256 the CR and CNR of opti256 are respectively 0.8 dB and 0.6 dB better.

Although opti256 performs the best in terms of axial resolution and has a small contrast improvement, the lateral resolution is 1.1 mm (+60%) worse than that obtained with rand256. The resolution capability of rand256 is on its side identical to the one of ref1024 (the scatterers look clearly identical). So from both a qualitative and quantitative point of view, the results of the 3-D DW mode images shows that a better compromise between resolution and contrast is yielded by rand256 compared to opti256 which invalidates the initial hypothesis that an array optimized for focused imaging could be optimal also for DW imaging (as further discussed in III).

## Discussion

This paper has shown that 3-D US images with good overall quality can be obtained with 2-D sparse arrays in both focused and DW modes. Of course, compared to the full array, rand256 and opti256 show an unavoidable sensitivity reduction since the active surface of the sparse arrays is reduced by 75%. Indeed, as measured on the central lines of the images shown in Fig. 2 the average RMS background values of opti256 and rand256 were respectively 17.8 dB and 24.0 dB below the one of ref1024 which can be theoretically grounded considering the following: in transmission (TX), when reducing the active surface by a factor of two a −6 dB sensitivity drop is expected (in fact the pressure is correspondingly halved). In reception (RX), only a −3 dB sensitivity drop is expected because signals constructively interfere, while input noise does not. So, we expect at least a theoretical 12 + 6 = 18 dB degradation while comparing a 256-element sparse array with a 1024-element array (with the same TX voltage applied in both cases). This estimate is consistent with the results obtained with opti256. Considering rand256, further beamforming degradation could be due to a worse spatial sampling which keeps the noise correlated.

However, the overall resolution and contrast performance is (in both focused and DW modes) competitive enough to produce images comparable to the ones produced by the reference ref1024 array. As expected rand256 and ref1024 yield the best resolution performance as their active elements are spread over the entire available surface. On the contrary, the opti256 array has active elements concentrated in the central part of the array (and no elements in the corner areas): this improves the array contrast capability (compared to rand256) since a “density tapering” contributes to lowering the sidelobes^42^. Moreover, in II.A (focused mode), it is shown that the ref1024 array has a slightly coarser resolution compared with the rand256. As the aperture size of ref1024 cannot be lower than the aperture size of rand256 no strong conclusions can be taken on the superiority of rand256 against ref1024 in terms of resolution.

The results of section II.A (focused mode) can be compared to the results of section II.B (DW mode), obtained with the same phantoms. In general, compounded DW mode seems to work better than the focused mode. Indeed, the reference full array (ref1024) demonstrates improvements in terms of both resolution and contrast (lateral resolution improved by 0.5 mm, CR improved by 1.3 dB, CNR improved by 0.5 dB) we used in DW mode. Notably, the DW mode improves the CR by 1.7 dB, 1.9 dB, and 1.3 dB for rand256, opti256, and ref1024 arrays, respectively. The DW mode also improves the CNR by 3.4 dB, 3.8 dB, and 0.5 dB for rand256, opti256, and ref1024 arrays, respectively. Furthermore, the 3-D DW mode is much more convenient in terms of volume rate: for a same PRF, the DW volume rate would be 36 times higher (90 volumes/second) than that obtained in focused mode. While rand256 shows a 0.4 mm lateral resolution improvement, in DW mode, the opti256 array performs better in focused mode rather than in DW mode (0.6 mm coarser lateral resolution in DW mode). This looks consistent with the fact that the sparse array optimization was obtained in focused mode^47^.

About the image texture, the sSNR value is 0.3 lower (on average) with DW compared to focused mode, which corresponds to an increased speckle variation (less homogeneous texture). Possibly, the DW pixel-wise reconstruction allows a larger variability in the speckle (which lowers the sSNR) while the scan lines are maintaining a relatively higher homogeneity along each line.

The choice of setting the virtual sources at 25 mm far from the array center represents a compromise between multiple imaging objectives. In theory, they should be located at 10 mm distance to produce a theoretical field of view of about ±30° but the image contrast would be significantly lower, as discussed in^4^. On the other hand, considering that the sparse array optimization was based on a TX focus at 40 mm, such depth could have been chosen, but in this case, the field of view would have been narrowed by ±7.6°. We thus chose an intermediate focal point at 25 mm also supported by 3-D simulations^47^, which showed a regular acoustic beam behaviour of the −6 dB iso-surface for depths between 18 and 50 mm. An inherent weakness of our choice is that the lateral portion of the images is not very good. The wires of the side grapes tend to disappear in the opti256 array (in focused mode, Fig. 1), but to a certain extent the overall image quality (in particular in terms of contrast) remains acceptable.

Finally, the hypothesis of optimally resolving virtual sources thanks to 2-D arrays optimized for focused imaging has not been fully validated. Indeed, during the optimization, even though the beam shape is controlled over 360° at several depths in focused mode^47^ (thus spontaneously yielding layouts with circular symmetry), there is no direct control on the homogeneity of the transmitted diverging wave front. The simulations presented in^47^ have assessed the full 3-D imaging capability in focused mode (in other slices than the XZ and YZ-orthogonal planes) but the full-3D DW behaviour (in arbitrary slicing planes) associated to the same array remains to be studied. Nevertheless, the current study has brought some insights on how changing the 2-D sparse array configuration impacts the quality of the 3-D DW US images compared to the 3-D focused mode (at least in the XZ and YZ-orthogonal planes): when the array shows a better resolution capability in focused mode, it shows general better behaviour in DW mode. So the ability for an array to transmit a suitable DW seems more connected to the ability of this array to resolve well the virtual source from which it propagates and less to cancel its potential virtual lateral neighbors (lateral lobes). So to “well represent virtual sources”, our study preferably suggests that the resolution capabilities of the array is more important than the lateral lobes constraint. In other words, these results suggest that to design an optimized 2-D sparse array dedicated to 3-D DW imaging, the priority should be given to a strong resolution constraint instead of a very low lateral lobes constraint. This of course requires further investigations to be properly integrated inside our optimization tool and the design of the right cost function will take advantage of the presented work to be defined (for example by considering different arrays for transmission and reception, the first one satisfying strong resolution constraints to help representing the virtual source correctly).

In this work, 3-D US images were obtained with two different 2-D sparse array configurations in both focused and diverging wave modes. Focused and DW acquisitions on the same phantoms allowed to compare the respective results. It was experimentally shown that good quality 3-D ultrafast US images can be produced by using diverging waves outperforming the focused imaging mode. The results also experimentally confirmed that a 2-D sparse configuration optimized for focused imaging^43^ can actually produce good quality images and, compared to a random array with the same number of elements, yields an improved trade-off between resolution and contrast. Furthermore, the comparison between focused and DW modes suggests some guidelines on whether to choose focused or DW mode, and in particular it suggests how the optimization should be specifically tuned to design 2-D sparse arrays dedicated to perform in DW mode. In conclusion, this work showed that 2-D sparse arrays are an effective and straightforward solution to perform 3-D ultrasound imaging by driving a reduced number of elements of a commercial 2-D array maintaining the opportunity to access continuously to all the active elements. Future investigations may consider reducing the number of active elements down to 64 (or even less!^60^) to implement ultra-light scanners and this issue could be addressed considering the element size as a new degree of freedom in the optimization to compensate for the extreme sensitivity loss^61^.

## Methods

### 2-D sparse arrays optimization for focused mode and hypothesis for DW virtual sources representation

#### Focused mode with random and optimized 2-D sparse arrays

There is no unified approach to design 2-D random sparse arrays but two main streams can be identified: (1) the classic “random” approach, in which the solution yielding the best performance (e.g., narrowest main lobe width or lowest lateral lobe level) is selected by an energy function computed only after the exploration process^45,49,62–64^; (2) the “stochastic” optimization approach, in which the exploration is led by a monochromatic^41–43,46,65–69^ or wideband^47,70^ energy function computed during the exploration process. The advantage of the second approach are the global minima convergence property and the finite time convergence property allowing both escaping from local minima and controlling the optimization process duration^53^.

In this work, two 256-element 2-D sparse arrays have been experimentally tested by selecting the elements out of a commercial 32 × 32 array probe. The selection was based on: a) a classic random approach (rand256); b) the stochastic optimization tool presented in^47^ (opti256). To allow a fair cross comparison between the results of rand256 and opti256, both exploration processes were setup to have the same state space (the set of all possible combinations of 256 active elements in a 32 × 32 array), the same mechanism to propose new candidates (one element random translation in its available 8-nearest neighborhood), the same energy function (beam pattern with a narrow main lobe and the lowest achievable lateral lobes at several depths) and the same computation effort (1 280 000 trials). During the optimization of opti256, “any state could be reached from any other state in a finite number of moves”^71^, so the classic random exploration process was also set to allow several visits of the same configuration. The only difference to obtain rand256 and opti256 was the unconditional acceptation of the new candidates in the case of rand256 whereas for opti256 the new candidates had a conditional probability to be accepted, which was decreasing with the number of trials. To sum up, rand256 and opti256 are the 256-elements arrays selected, by the same energy function, as the “best” solution encountered during two different explorations (classic random and stochastic optimization) made of 1 280 000 trials.

#### Diverging wave mode with multiple virtual sources

The 2-D sparse arrays that were tested in DW imaging mode are the same arrays that were described above for focused imaging (IV.C.1). In particular, the optimized 2-D sparse array (opti256) was shown capable of producing a focused beam with lateral lobes as low as possible. According to the time reversal principle^72^, if an array can produce, in front of itself, a focal spot $({x}_{{\rm{f}}},\,{y}_{{\rm{f}}},\,{z}_{{\rm{f}}})$ clear of artifacts, it should also reproduce, behind itself, a virtual source $({x}_{{\rm{f}}},\,{y}_{{\rm{f}}},\,-\,{z}_{{\rm{f}}})$ with no artifact. For this reason, in the present work, the 2-D sparse arrays optimized for focused mode (opti256) were supposed relevant candidates to produce artifact-free virtual sources for 3-D DW imaging. The rand256 and the reference full array (ref1024) were thus used to produce 3-D DW images, too.

### 2-D sparse array implementation by weighting a fully populated 1024 elements matrix

**Figure 4:**
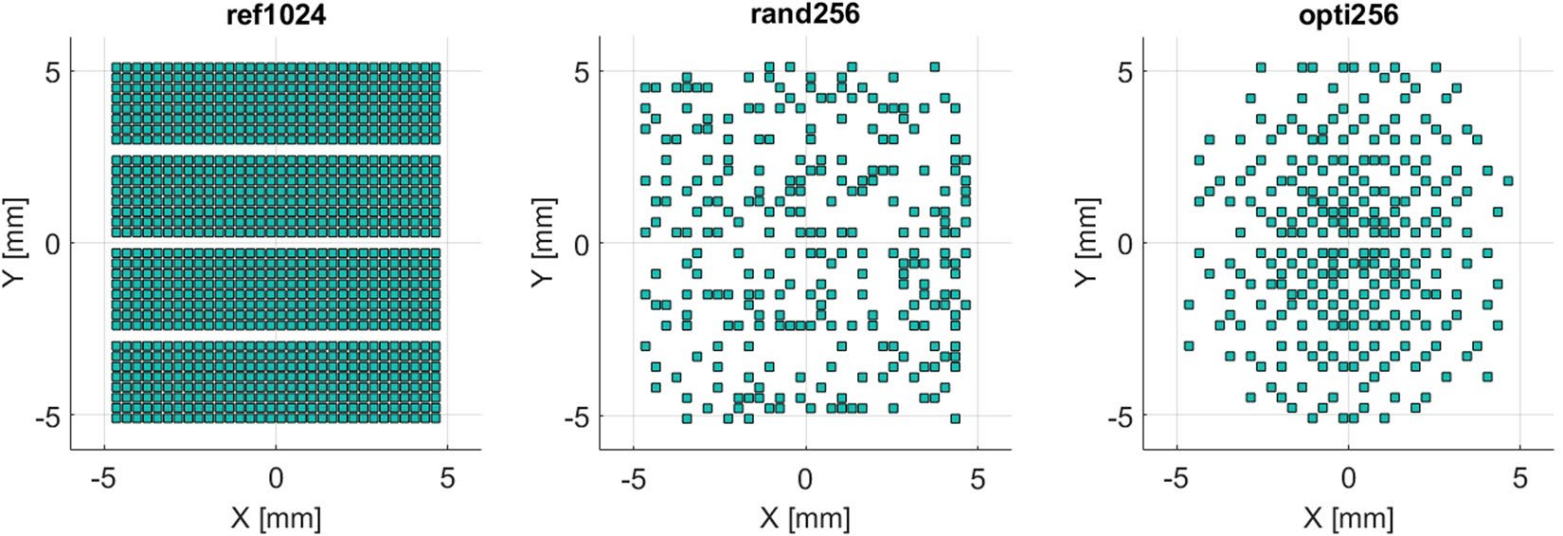
Illustration of the selected layouts: the reference array (ref1024), the random array (rand256), and the optimized array (opti256).

Four Vantage-256 research scanners (Verasonics, Inc., Kirkland, WA, USA) working at 12 MHz sampling frequency were synchronized to drive up to 1024 elements of an array^24^. They were connected to a 32 × 32 element probe (Vermon, Tour, France) having 72% bandwidth at 3 MHz and a square footprint with sides of about 10 mm. The element size is 249 μm and the pitch 300 μm in both the *x*- and *y*-directions. Each element was physically connected to the same scanner channel both in transmission and reception (one-element-to-one-channel design). In total, three 2-D array configurations were implemented (the layouts are shown in Fig. 4.): the full 32 × 32 reference array (ref1024), the random (rand256) and optimal (opti256) 2-D sparse arrays configurations described in IV.A.1.

In practice, a different weighting map for the 1024 elements was associated to each array configuration: the elements/channels weighted with “one” were active, whereas those weighted with “zero” were de-activated. As a result, for each configuration, only the active elements were excited during transmission and only their echoes recorded during reception. Except this binary weighting map, no other apodization was used.

### Experimental set-up and imaging strategies

#### Focused wave imaging

The same imaging sequence focused at 25 mm depth was adopted for all the considered 2-D arrays described in IV.A.1 (ref1024, rand256, and opti256). The synchronized ultrasound scanners (described in IV.B) were programmed to transmit 3-cycle 3 MHz sine bursts. The maximum depth of interest was set at 130 mm and the PRF was set at 2250 Hz, producing a volume rate of 2.5 Hz. A summary of the common parameters that were used for all of the acquisitions are listed in Table 3. In reception, each Verasonics scanner recorded the radiofrequency (RF) signals from the active elements to which it was connected to, and this was repeated for all scan lines. After the acquisition, the entire volume dataset was concatenated by grouping the four partial data located on each scanner. A dataset for an entire volume was made of 2048 16-bit samples for each RF line, to be multiplied by 31 × 29 = 899 scan lines (Table 3). Thus, in the case of ref1024, the dataset size is 3.8 GB, while in rand256 and opti256 it is directly divided by four. The volume reconstruction was performed applying the delay-and-sum algorithm to the RF data set.

**Table 3:** Experimental parameters.

**Acquisition parameters**	
*TX Excitation signal*	3-cycle sine @3MHz without temporal weighting
*RX Sampling frequency*	12 MHz
*Grayscale phantoms*	Gammex (Sono410 SCG), CIRS (054GS)
*Acquisition depth range*	15 mm to 130 mm
*Pulse repetition frequency (PRF)*	2250
**Transducer parameters**	
*Central frequency*	3 MHz
*Bandwidth (−6 dB)*	72 %
*Aperture size*	9.6 × 10.5 mm^2^ (~20$\lambda$)
*Element size (squares)*	249 µm
*Pitch*	300 µm
*Elements apodization*	1 (activated) or 0 (deactivated)
**Imaging parameters (Focused waves)**	
*Focal distance*	25 mm from the array center
*Sector scan range*	±30° in both elevation and azimuthal directions
*Number of scan lines*	azimuthal × elevation*:* 31 × 29
*Reconstruction depth range*	15 mm to 130 mm
*Volume rate*	2.5 volume/second
**Imaging parameters (Diverging waves)**	
*Virtual sources distance*	25 mm from the array center
*Sector scan range*	±30° in both elevation and azimuthal directions
*Virtual sources angle range*	±15° in both elevation and azimuthal directions
*Reconstruction depth range*	15 mm to 90 mm
*Compounding*	25 DWs/volume
*Volume rate*	90 volumes/second

#### Diverging wave imaging

**Figure 5:**
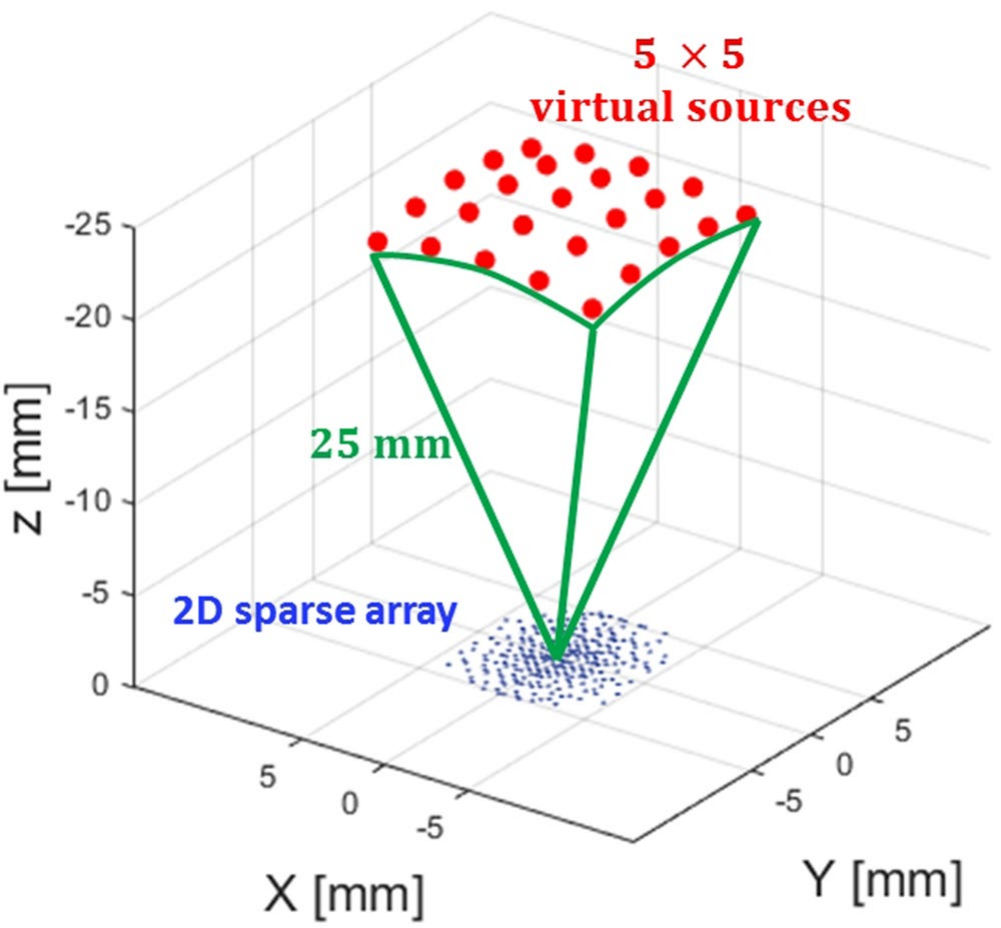
Illustration of the 25 virtual sources used to transmit diverging waves by means of 2-D sparse arrays. The virtual sources are located behind the array at a distance of 25 mm from the array centre.

As in focused mode, the four Vantage-256 systems were synchronized to drive the active elements of the array in DW mode. The same DW imaging acquisition sequence was performed for the three tested arrays (ref1024, rand256, and opti256). Multiple volumes were acquired by using 5 × 5 virtual sources distributed over a spherical cap at 25 mm distance from the center of the array (Fig. 5). Such distribution yields a theoretical field of view of ±25.4°, which is close enough to the ±30° aperture used in focused mode to permit a comparison although a small darkening of the DW image sides has to be expected. As in focused mode, the systems were programmed to transmit 3-cycle 3 MHz sine bursts from each active element. The reconstruction of each DW volume was performed from 15 mm to 90 mm with a delay-and-sum algorithm applied to the raw RF signals acquired after each transmitted DW. The final volume was obtained by averaging the datasets related to the 25 virtual sources to form a 25-DW-compounded volume with enhanced contrast and resolution. The number of virtual sources was chosen to be odd (ensuring a central virtual source above the array) and represents a reasonable trade-off between 3 × 3 (insufficient contrast improvement) and 7 × 7 (that would divide the volume rate by a factor of two). The PRF was set at 2250 Hz allowing an acquisition rate of 90 volumes per second.

### Phantoms and evaluation criteria

In the experiments, the Gammex (Sono410 SCG) and the CIRS (054GS) US phantoms were used. For each phantom, acquisitions in both focused and DW modes were performed, by taking care that neither the probe nor the phantom was moved between the two acquisition sets.

**Figure 6:**
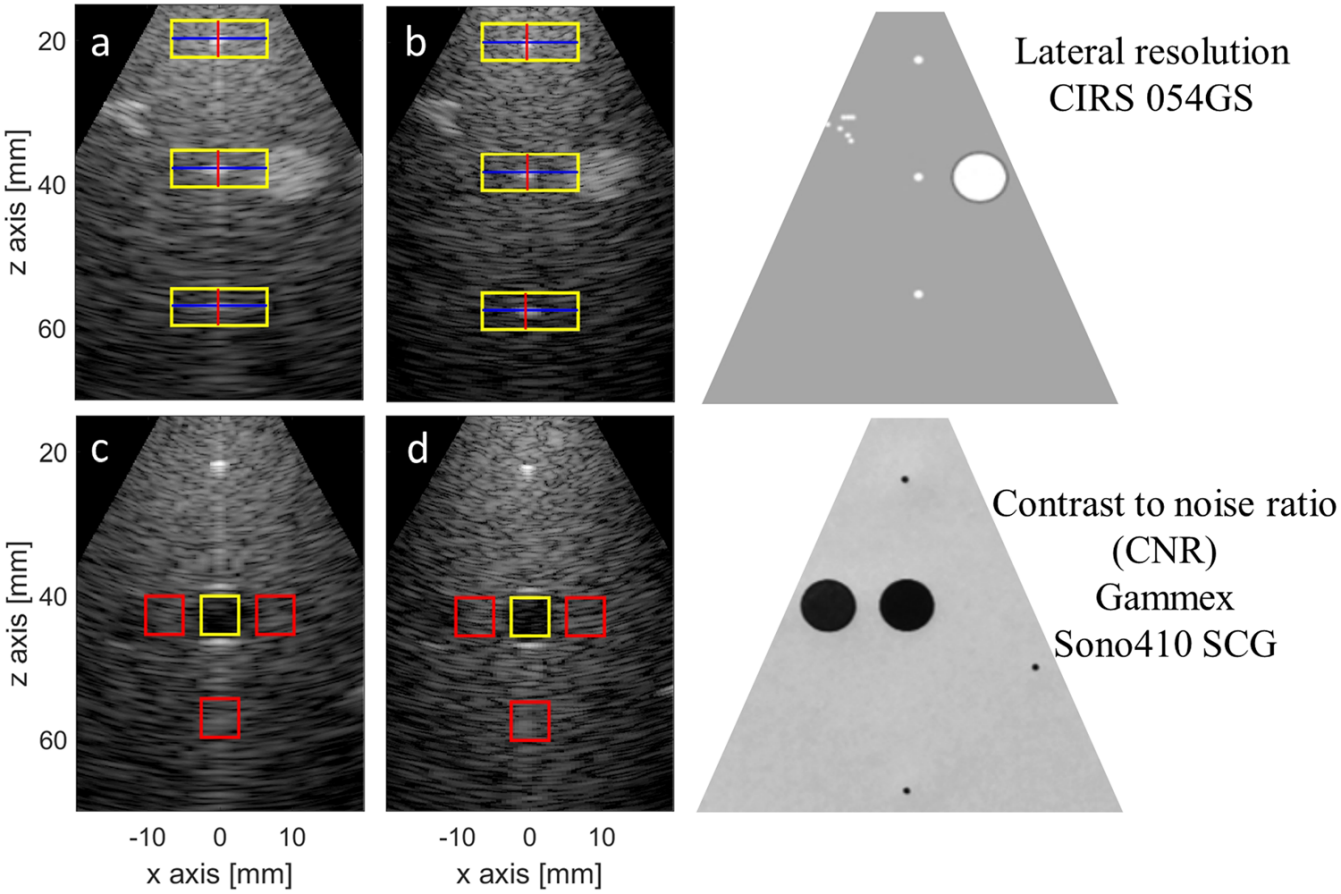
Regions of interest used to estimate the lateral and axial resolutions (**a**,**b**) on the CIRS (054GS) phantom and (**c**,**d**) the CNR on the Gammex (Sono410 SCG) phantom (the two phantom structures are displayed on the right). The regions are shown for the focused (**a**,**c**) and the diverging wave (**b**,**d**) modes. For the CNR evaluation the region inside the cyst was delimited by the yellow square of diagonal 7.4 mm and the three different background regions are drawn as red squares (same size as the yellow one).

The image quality was assessed using similar metrics as in the PICMUS challenge^73^: the comparison criteria were the lateral and axial resolution, the contrast ratio (CR), the contrast-to-noise ratio (CNR), and the speckle signal-to-noise ratio (sSNR). These parameters were measured on the XZ slice images obtained by scanning the CIRS (054GS) for the resolution and the Gammex (Sono410 SCG) phantoms for the other metrics. The resolution metrics were computed through the full width at half maximum (FWHM) of the point spread functions corresponding to the three central wires located at depths 20, 40 and 60 mm in the CIRS phantom. With reference to Fig. 3, the −6 dB lateral and axial resolution were evaluated on the profiles extracted in correspondence of the horizontal (blue) and vertical (red) lines crossing at the pixel of maximum intensity in the local region of interest (ROI, represented by the yellow rectangles in Fig. 6a,b). The CR, CNR and sSNR metrics were respectively computed according to:

1 $${\rm{CR}}=20\,{\mathrm{log}}_{10}(\frac{{\mu }_{{\rm{in}}}}{{\mu }_{{\rm{out}}}})$$

2 $${\rm{CNR}}=20\,{\mathrm{log}}_{10}(\frac{2\,|{\mu }_{{\rm{in}}}-{\mu }_{{\rm{out}}}|}{\sqrt{({\sigma }_{{\rm{in}}}^{2}+{\sigma }_{{\rm{out}}}^{2})}})$$

3 $${\rm{sSNR}}=\frac{{\mu }_{{\rm{out}}}}{{\sigma }_{{\rm{out}}}}$$

where ${\mu }_{{\rm{in}}}/{\mu }_{{\rm{out}}}$ and ${\sigma }_{{\rm{in}}}/{\sigma }_{{\rm{out}}}$ correspond to the respective mean and standard deviation of the beamformed signals envelope (before log-compression) values inside/outside the cyst: the inner region was delimited by a square of diagonal 7.4 mm (8 mm diameter cyst) and three different outer regions were considered: ROI 1 is on the left side of the cyst, ROI 2 is on the right side of the cyst and ROI 3 is below the cyst (red squares on Fig. 6). So the contrast metrics were computed three times and averaged to avoid undesired signal attenuation or random speckle noise effect on the measurements. Note that the outer regions were squares of same area as the inner region. For each metrics, the regions used for the evaluation were identical, as shown on Fig. 6, for both the focused and DW images and the same probe position was used for all the acquisitions made on the same phantom to enable a fair comparison between the two imaging strategies.

From the indicators definition, the following general interpretation can be inferred: good resolution is associated with low FWHM values, good contrast is indicated by large negative values of CR values (anechoic cyst) and large positive (or at least less negative) values of CNR, and good background homogeneity implies high sSNR amplitudes (large positive values). The evaluation results are reported in Table 1 for the focused mode and in Table 2 for the DW mode.

### Data availability

The datasets generated during and analysed during the current study are available from the corresponding author on reasonable request.
